# Caffeine improves reaction time, vigilance and logical reasoning during extended periods with restricted opportunities for sleep

**DOI:** 10.1007/s00213-014-3834-5

**Published:** 2014-12-21

**Authors:** Gary H. Kamimori, Tom M. McLellan, Charmaine M. Tate, David M. Voss, Phil Niro, Harris R. Lieberman

**Affiliations:** 1 Behavioral Biology Branch, Center for Psychiatry and Neuroscience, Walter Reed Army Institute of Research, BLD 503, Silver Spring, MD 20910 USA; 2 DRDC Toronto, 1133 Sheppard Avenue West, Toronto, ON M3M 3B9 Canada; 3 New Zealand Defence Force, Auckland, New Zealand; 4 Military Nutrition Division, US Army Research Institute of Environmental Medicine (USARIEM), Natick, MA 01760-5007 USA; 5 TM McLellan Research Inc., 25 Dorman Drive, Stouffville, ON L4A8A7 Canada

**Keywords:** Sleep loss, Sustained operations, Afternoon sleep, Cognitive function, Marksmanship

## Abstract

**Rationale:**

Various occupational groups are required to maintain optimal physical and cognitive function during overnight periods of wakefulness, often with less than optimal sleep. Strategies are required to help mitigate the impairments in cognitive function to help sustain workplace safety and productivity.

**Objectives:**

To test the effectiveness of repeated 200 mg doses of caffeine on cognitive function and live-fire marksmanship with soldiers during three successive nights of sustained wakefulness followed by 4-h afternoon sleep periods.

**Methods:**

Twenty Special Forces personnel (28.6 ± 4.7 years, 177.6 ± 7.5 cm and 81.2 ± 8.0 kg) were randomly assigned to receive four 200-mg doses of caffeine (*n* = 10) or placebo (*n* = 10) during the late evening and early morning hours during three successive days. An afternoon 4-h sleep period followed. The psychomotor (PVT) and field (FVT) vigilance, logical reasoning (LRT) tests and a vigilance monitor assessed cognitive function throughout the study. Live-fire marksmanship requiring friend–foe discrimination was assessed.

**Results:**

Caffeine maintained speed on the PVT (*p* < 0.02), improved detection of events during FVT (*p* < 0.001), increased number of correct responses to stimuli as assessed by the vigilance monitor (*p* < 0.001) and increased response speed during the LRT (*p* < 0.001) throughout the three overnight testing periods. Live-fire marksmanship was not altered by caffeine.

**Conclusions:**

A total daily dose of 800 mg caffeine during successive overnight periods of wakefulness is an effective strategy to maintain cognitive function when optimal sleep periods during the day are not available.

## Introduction

Many occupational groups, such as shift workers, long-haul truck drivers, emergency responders and deployed military personnel, must maintain optimal cognitive and physical performance over several consecutive days; often, this occurs with inadequate sleep opportunities provided at non-optimal periods during the day. Under these conditions, strategies to counter the impairments in cognitive function that accompany sleep loss (Belenky et al. 2003; Lieberman et al. 2002, 2005; Thomas et al. 2000) are needed to help sustain workplace safety and productivity. Furthermore, appropriate countermeasures need to be developed based on both controlled laboratory and field experimentation.

Caffeine (1,3,7 trimethylxanthine) is the most widely consumed psychoactive substance in the world (Barone and Roberts 1996). When consumed, it is absorbed and distributed throughout the body, including the brain, and exerts its influence as an adenosine A_1_ and A_2A_ receptor antagonist (for reviews, see Carvey et al. 2012; Fredholm et al. 1999). Caffeine has significant effects on arousal of rested individuals (Lieberman et al. 1987; Smith et al. 1999; Warburton 1995) and substantial positive effects on vigilance, alertness and mood during periods of sleep loss (Kamimori et al. 2000, 2005; Lieberman et al. 2002). A relatively high 600-mg dose of caffeine is as effective as prescription medications such as modafinil and amphetamines for improving cognitive function and countering sleep loss during periods of prolonged wakefulness (Wesensten et al. 2002, 2004, 2005a). Daily consumption of caffeine is considered safe and without long-term risk for healthy adults (Bordeaux and Lieberman 2013; Higdon and Frei 2006; Nawrot et al. 2003). Thus, caffeine is a viable candidate to sustain productivity and safety in the workplace during periods of extended operations that restrict sleep.

Military operations are an occupational setting where soldiers can be required to maintain periods of prolonged wakefulness, which can lead to extensive decrements in cognitive function (Belenky et al. 1994; Lieberman et al. 2005). Lieberman et al. (2002) studied the ability of caffeine to maintain cognitive function during a stressful military training exercise, which involved more than 3 days of sleep restriction together with physical and mental challenges. Following 72 h of sleep deprivation, a 200- and 300-mg dose of caffeine improved several measures of cognitive function including vigilance, reaction time, attention and mood, as well as improving speed of target acquisition during marksmanship testing compared with placebo (Lieberman et al. 2002; Tharion et al. 2003). A subsequent series of studies collectively demonstrated that a total dose of 600 mg caffeine provided throughout a night without sleep was effective in maintaining vigilance during both simulated urban and field observational tasks (McLellan et al. 2005a, b), restoring or improving physical performance compared with the rested state (McLellan et al. 2004, 2005b), as well as improving or maintaining marksmanship (McLellan et al. 2005a; Tikuisis et al. 2004).

Although caffeine’s positive effects on vigilance and arousal during periods of sustained wakefulness have been repeatedly demonstrated (Beaumont et al. 2001; Bonnet et al. 1995; Kamimori et al. 2005; Lieberman et al. 2002; McLellan et al. 2005a, b), the effect of the drug on higher level cognitive function, such as executive control of attention and working memory, during periods of sleep deprivation appears less consistent (Wesensten et al. 2005a). Cognitive demands in a military workplace often require making complex decisions and responding and adapting to novel situations. Thus, to demonstrate caffeine’s effectiveness in such an occupational setting, it is necessary to assess the efficacy of caffeine on more complex tasks, in addition to tests of vigilance and reaction time.

Furthermore, restricting sleep periods to the daytime over several days is a scenario consistent with current military engagements and demands of other occupations that require shift work. Often, these daytime sleep periods are not of sufficient duration or may not provide sleep of optimal quality (Torsvall et al. 1989). Thus, it is not known whether these work conditions might increase the need to use a counter-measure strategy, such as caffeine, to help sustain cognitive function. Previous studies that have assessed the use of caffeine and afternoon sleep periods have used the afternoon sleep as a preventative measure prior to a period of overnight sustained wakefulness (Bonnet and Arand 1994b; Bonnet et al. 1995; Schweitzer et al. 2006). The authors are unaware of studies that have compared the effectiveness of recovery afternoon sleep periods on performance with or without caffeine during subsequent overnight periods of sustained wakefulness.

The purpose of this study was to examine the effectiveness of repeated administration of 200 mg doses of caffeine to sustain vigilance, reaction time, higher-order cognitive function that requires logical reasoning and live-fire marksmanship during three successive evenings of sustained wakefulness followed by 4-h afternoon periods for sleep. Caffeine was delivered in gum form since it is absorbed more rapidly into the circulation, presumably through the buccal tissue, than caffeine ingested in pill form (Kamimori et al. 2002). Furthermore, this mode of delivery was chosen to be consistent with our previous studies that assessed the physical and cognitive effects of caffeine during sustained military operations conducted overnight (McLellan et al. 2005a, b). It was hypothesised that the cognitive function and marksmanship of participants receiving caffeine would be enhanced compared to those receiving placebo, despite the opportunity for all volunteers to sleep during the afternoon periods.

## Methods

### Participants

This study was approved by the human ethics review committees of Walter Reed Army Institute of Research USA, Defence R&D Canada–Toronto, the New Zealand Northern Region and the New Zealand Defence Force. Volunteers were not enrolled if they were taking any medication or if they had given blood within 30 days of the study. Twenty male Special Forces personnel aged 28.6 ± 4.7 years (mean ± SD), with height of 177.6 ± 7.5 cm and body mass of 81.2 ± 8.0 kg participated in this study. All were cleared to participate in the study by their base Medical Officer. An initial briefing informed potential volunteers of the details, discomforts and risks associated with the experimental protocol, and written informed consent was obtained. After the briefing, soldiers were assigned randomly into five groups of four with two soldiers in each group receiving caffeine (total *n* = 10) or placebo (total *n* = 10) in a double-blind manner. The soldiers were informed that no coffee- or caffeine-containing products were to be consumed after awakening on the morning of day 1 of the study. The limited number of volunteers precluded inclusion of additional caffeine and placebo groups that would not be given the opportunity for daytime sleep. However, since current military operational scenarios provide limited sleep opportunities during the day, the current study was designed to be of the greatest ecological validity for the end-user.

### Caffeine questionnaire

A questionnaire was administered to determine average daily caffeine consumption. This questionnaire collected information regarding the type and volume of caffeinated beverages consumed daily, such as coffee, tea, soft drinks and energy drinks, daily consumption of chocolate bars and candies and the use of caffeinated over-the-counter medications. The calculated average caffeine use for each individual was used to examine the relationship between caffeine use and field vigilance test (FVT) performance.

### Caffeine and placebo

Caffeine or placebo was administered at 2145, 0100, 0345 and 0700 h beginning the evening of day 2 and continuing through the morning of day 5 (Table 1). Two pieces of 100-mg caffeine gum (Stay Alert®, Marketright Inc., Plano, IL) or placebo were chewed for 5 min for a total dose of 800 mg caffeine over a 24-h period. This total overnight dose of 800 mg caffeine was greater than used during previous studies that examined a single overnight period of sustained wakefulness and testing (McLellan et al. 2005a, b). However, this repeated 200 mg dose of caffeine at 2–3-h intervals is effective for sustaining elevated plasma caffeine levels and maintaining cognitive performance throughout a night of sleep loss (Kamimori et al. 2005; Syed et al. 2005). In addition, the last 200 mg dose in the morning was provided in close proximity to the performance of an obstacle course. Both the placebo and caffeine gum contained sugar, gum base, corn syrup, natural and artificial flavours, fructose, glycerine, sucralose, artificial colour and butylated hydroxytoluene and were similar in appearance and taste.

**Table 1:** The timeline for the study design

Day	Time (h)	Task
One	0745	Participant briefing and initial training on cognitive tasks
	0815	Control live-fire and familiarization of obstacle course
	2000	Familiarization of cognitive tasks
	2030	Control FVT
	2230	Familiarization of cognitive tasks
Two	0800	Control obstacle course
	0900	Control cognitive tasks
	1815	PVT, LRT
	2100	PVT, LRT
	2145	200 mg drug or placebo, PVT
	2200	Live-fire marksmanship followed by PVT
Three	0045	PVT, LRT
	0100	200 mg drug or placebo, PVT
	0130	FVT session 1
	0330	PVT, LRT
	0345	200 mg drug or placebo, PVT
	0415	FVT session 2
	0615	PVT, LRT
	0700	200 mg drug or placebo, PVT
	0730	Obstacle course
	1300	PVT followed by 4-h sleep period
	1815	PVT, LRT
	2100	PVT, LRT
	2145	200 mg drug or placebo, PVT
	2200	Live-fire marksmanship followed by PVT
Four	0045	PVT, LRT
	0100	200 mg drug or placebo, PVT
	0130	FVT session 3
	0330	PVT, LRT
	0345	200 mg drug or placebo, PVT
	0415	FVT session 4
	0615	PVT, LRT
	0700	200 mg drug or placebo, PVT
	0730	Obstacle course
	1300	PVT followed by 4-h sleep period
	1815	PVT, LRT
	2100	PVT, LRT
	2145	200 mg drug or placebo, PVT
	2200	Live-fire marksmanship followed by PVT
Five	0045	PVT, LRT
	0100	200 mg drug or placebo, PVT
	0130	FVT session 5
	0330	PVT, LRT
	0345	200 mg drug or placebo, PVT
	0415	FVT session 6
	0615	PVT, LRT
	0700	200 mg drug or placebo, PVT
	0730	Obstacle course
	0920	PVT followed by 2-h sleep period
	1130	PVT, de-brief and release

Cognitive tasks included logical reasoning (LRT), psychomotor vigilance (PVT) and the field vigilance (FVT) tests

### Study design

The study timeline is presented in Table 1. After reporting for duty on the morning of day 1, participants were familiarised with the cognitive tasks and monitor, which included the psychomotor vigilance test (PVT), the logical reasoning test (LRT) and the wrist-worn vigilance monitor, described below. They also performed a familiarization trial on the obstacle course and control live-fire marksmanship testing. Results from the obstacle course are provided elsewhere (McLellan et al. 2007). After conducting regular training exercises for the remainder of the day, additional practice on the cognitive test battery and control testing for the FVT were conducted in the evening. A normal 8-h period of sleep was provided overnight between days 1 and 2.

After reporting for duty on the morning of day 2, soldiers performed control baseline testing for the obstacle course and the cognitive tasks. Regular military training was conducted for the remainder of the day. The experimental period began after an evening meal. Participants were tested through the evening of day 2 and morning of day 3 and did not have an opportunity for sleep until 1330 hrs on day 3 for a 4-h period (Table 1). Participants repeated the same testing throughout the evening of day 3 and morning of day 4 and were provided with another 4-h period for sleep at 1330 h of day 4. Regular meals were provided throughout, and participants conducted their normal training when their time was not occupied by experimental procedures. Testing was again repeated throughout the evening of day 4 and morning of day 5, with the trial ending at 1130 h on the morning of day 5 when participants were de-briefed and released (Table 1).

### Psychomotor vigilance test (PVT)

The PVT was administered on Handspring Palm Platinum personal digital assistants (PDAs) running Palm operating system 3.1. The PVT is a test of continuous vigilance (Dinges and Powell 1985). The test presents a visual cue (a bull’s-eye) that appears in the middle of a screen. The subject’s task is to press a designated key as soon as possible after the visual cue appears. The time between each presentation of the cue is randomised to between 1 and 5 s with a total of 85 stimuli presentations per test session. Performance is recorded on a minute-by-minute basis. Variables measured include reaction time, and the number of minor (a reaction time > 500 ms < 3000 ms) and major lapses (a reaction time ≥ 3000 ms) before responding to the cue. Speed is presented as the inverse of reaction time.

### Logical reasoning test (LRT)

The LRT was administered on Sony Clie PDAs using Palm operating system 5.0. This test is an adaptation of a pencil and paper linguistic task requiring knowledge of English grammar and syntax (Baddeley 1968) and the ability to determine whether various simple sentences correctly describe the relational order of two symbols. On each trial, the symbol pair “# &” or “& #” is displayed along with a statement directly under it that correctly or incorrectly describes the order of the symbols as depicted in the example below:

- & #
- # is first

The participant must decide as quickly as possible whether the statement is true or false and then press the corresponding response button. There were 32 statements presented. Dependent measures included the number correct and average correct mean response time (seconds).

### Vigilance monitor

The vigilance monitor is a lightweight (48 g) ambulatory monitoring system (VIGMON II, Precision Control Devices, Inc., Fort Walton Beach, FL), slightly larger than a wristwatch (3.8 × 3.8 × 1.2 cm) worn on the non-dominant wrist. The monitor continually assesses a variety of behavioural and environmental factors, such as temperature, humidity and sleep/wake cycles (Lieberman and Coffey 1997). Volunteers were instructed to respond to a sequence of up to three vibrating stimuli, similar to the vibration of a pager or mobile phone, during the 2-h FVT test sessions. During these intervals, the stimuli were presented randomly every 5 to 20 min, with an average inter-stimulus interval of 15 min. The volunteer was instructed to push a small button on the monitor in response to the stimulus.

Each sequence of stimuli provided up to three opportunities to respond. Once a subject responded, no additional stimuli were presented until the next sequence occurred. If the subject did not respond to the first stimulus in the sequence (lasting 120 ms, 40 % max vibration) within 6 s, a second slightly longer (160 ms) and more intense stimulus (50 % max) was presented, and the subject had another 6 s to respond. If he still failed to respond a third and final longer (240 ms) and more intense (60 % max) stimulus was presented and the subject had eight additional seconds to respond. If the subject responded to any of the stimuli in a sequence, it was recorded as a “hit.” If the subject did not respond at all, it was recorded as a “miss.” Data were analyzed as correct responses and latency to respond from the first stimulus offset.

### Actigraphy

Volunteers wore an actigraph (Octagonal Sleep Watch, Precision Control Design, Ft Walton beach, FL) on their non-dominant arm for 7 days prior to and throughout the study. The actigraph is a device approximately the size of a diving watch and records arm movement. Actigraphy records were downloaded to a computer and scored as either “sleep” or “wake” using an algorithm developed at the Walter Reed Army Institute of Research.

### Field vigilance test (FVT)

Details of this test have been provided previously (McLellan et al. 2005b). Briefly, participants assumed a seated or prone position 4–5 m apart, 175–200 m away from a building façade illuminated by interior and exterior lights. This façade was used regularly for the participants’ normal night vigilance training, and, as a result, they were familiar with the nomenclature used to identify windows, doors and floors on the building. The participants were required to record where, when and what of any activity that occurred in and around this building over a 120-min observational period. Within each 20-min block, one activity that lasted approximately 5 s was randomly presented. Each activity was awarded a maximum of 3 points, one for recording the appropriate time of the activity, one point for describing the activity accurately and a third point if the volunteer stated accurately where the activity occurred. The total possible points awarded for this activity was 18.

After each FVT session, participants were asked to record the amount of time they felt they were asleep. They were asked to place a mark on a scale delineated into 15-min blocks from 0 to 120 min.

### Live-fire marksmanship test

This live-fire room-clearing exercise consisted of three scenarios designed to evaluate friend–foe identification effectiveness under time pressure. Participants had to accurately distinguish cardboard picture targets of friends (hostages) from foes (terrorists). Dependent variables included the percentage of friends and foes correctly targeted during a trial.

### Statistical analyses

The data were analysed with an analysis of variance with one grouping factor (drug or placebo) and repeated factors for time represented as days and testing sessions within each day. All analyses were conducted using SPSS (version 15.0; SPSS Inc., Chicago, IL, U.S.A.). To correct for the violation of the sphericity assumption with the repeated factor, a Huynh-Feldt correction (Lyman 1993) was applied to the F-ratio. When a significant F-ratio was obtained, a least significant difference post hoc analysis was used to isolate differences among treatment means. Pearson-product correlational analyses were performed for both groups separately to determine the relationship between their history of caffeine use and changes from control scores in FVT performance during each overnight period throughout the trial. An average FVT score was determined during each night of testing and subtracted from the control value. For all statistical analyses, the 0.05 level of significance was applied. Values are presented as mean ± standard error of the mean.

## Results

Analysis of the caffeine-use questionnaire indicated that two participants receiving caffeine and three receiving placebo were nonusers of caffeine. For the remaining participants, routine caffeine consumption varied from a low of 5 to a high of 410 mg day^−1^. Average daily caffeine consumption was not significantly different between the caffeine (86.9 ± 27.1 mg day^−1^) and placebo (170.7 ± 43.3 mg day^−1^) groups. Although the mean values were not different, the placebo group had five low consumers of caffeine (less than 50 mg day^−1^) and two higher consumers (410 and 290 mg day^−1^), whereas the caffeine group had six low consumers and only one higher consumer (225 mg day^−1^).

### Wrist monitors and sleep patterns

On the evening immediately preceding the study, there was no difference between treatment groups in total sleep time (*p* > 0.05) assessed by wrist monitors. There was a significant main effect for group for the amount of sleep recorded during the afternoons on days 3 and 4, and the morning of day 5. Participants receiving caffeine (64.6 ± 16.8 min) slept significantly less than those receiving placebo (115.3 ± 15.0 min) during all three sleep opportunities.

Since participants remained essentially motionless for the duration of the FVT sessions, the wrist monitors were not able to discern sleep periods from times of minimal motion. As a result, these data are not presented. There was a main effect of group and session for self-reported sleep during the FVT, but no other effects or interactions were observed. The placebo group reported significantly more sleep (62.4 ± 7.2 min) than the caffeine group (19.8 ± 7.2 min) throughout each overnight FVT, and both groups reported less sleep during FVT session 1 (37.8 ± 4.8 min) than FVT session 2 (44.4 ± 5.4 min) each night.

### Psychomotor vigilance test (PVT)

**Fig. 1:**
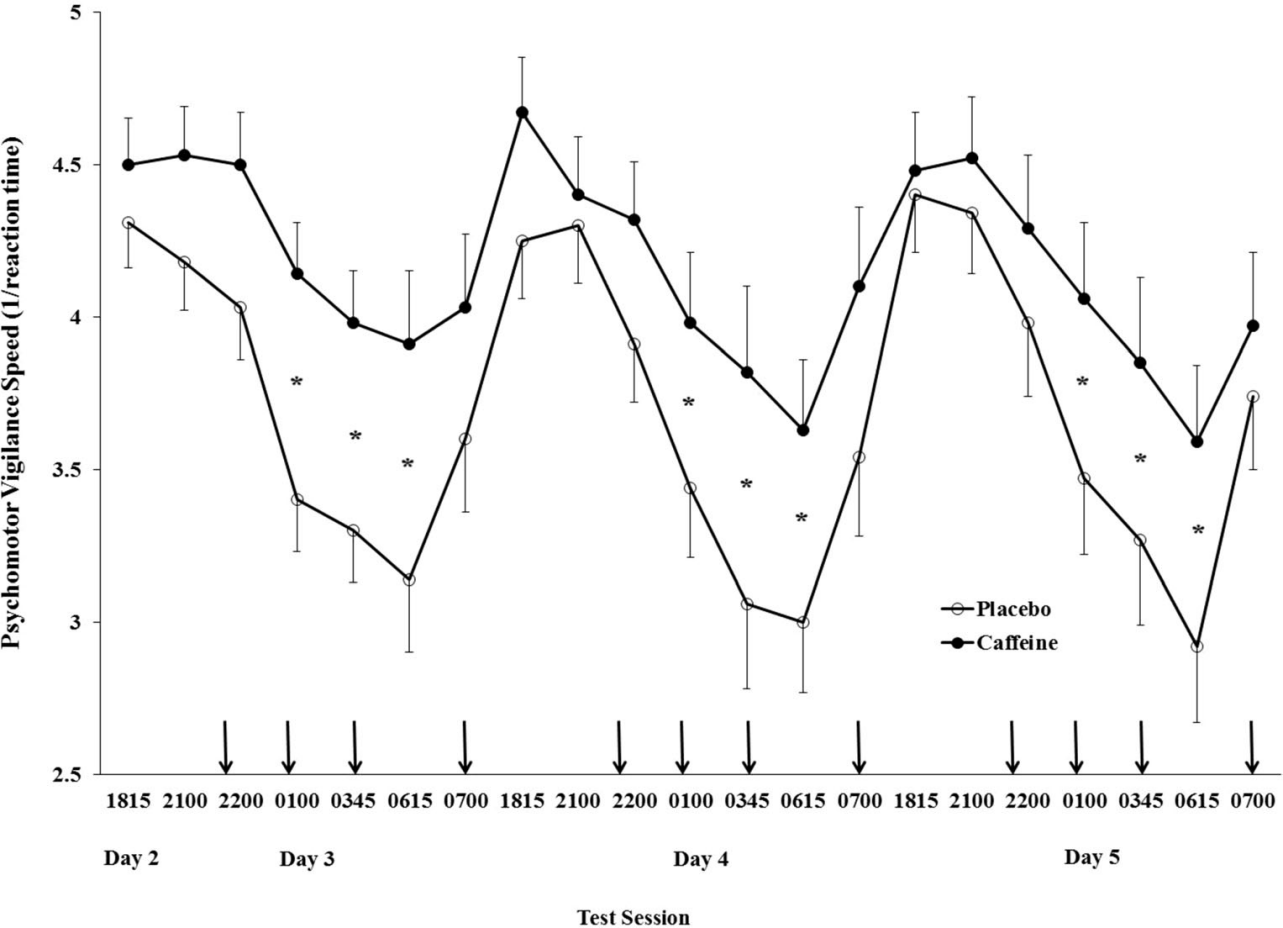
Response speed during the psychomotor vigilance test beginning during the evening of day 2 through to the morning of day 5 for the caffeine and placebo groups. The *asterisk* indicates a significant group by time interaction over the 3 days. The *arrows* indicate when 200 mg of caffeine or placebo was administered

There was a significant main effect for time (*p* < 0.001) and a significant group × time interaction (*p* < 0.02) for PVT. Participants in both groups were significantly slower on the PVT during the overnight testing beginning at 0045 until 0700 h compared with testing earlier in the evening. As shown in Fig. 1, there was no difference in PVT between groups at the beginning of each evening of testing. However, those receiving caffeine were significantly faster on the PVT than placebo at 0100, 0345 and 0615 h across days 3, 4 and 5.

There was no difference between groups in minor or major lapses on PVT. Participants in both groups had more major lapses at 0350 h on day 3 (0.35 ± 0.11) than at any other time or day.

### Field vigilance test (FVT)

**Fig. 2:**
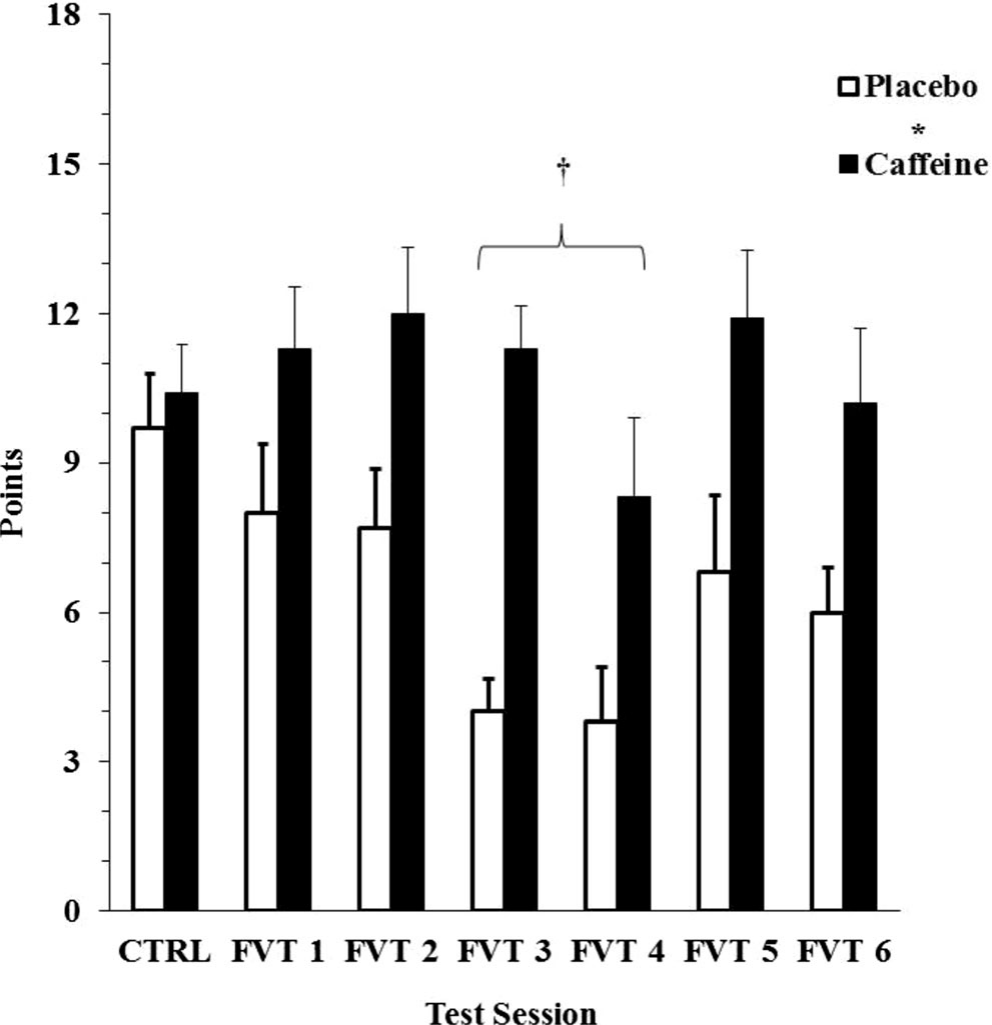
The number of points recorded during the control (CTRL) and six field vigilance test (FVT) sessions for the caffeine and placebo groups. The *asterisk* indicates a significant main effect between groups throughout the overnight testing sessions, whereas the *cross* indicates that both groups performed worse during the second night of testing

Preliminary findings from this test have been described previously (McLellan et al. 2007). Details of the control and overnight FVT scores are presented in Fig. 2. There was no difference between groups during the control testing but the caffeine group scored significantly more points (*p* < 0.001) than those receiving placebo throughout the three nights of restricted sleep (10.8 ± 1.4 versus 6.1 ± 0.8 points for caffeine and placebo, respectively). Both groups performed significantly worse (*p* < 0.001) during the second night of testing (6.9 ± 1.1) compared with night 1 (9.8 ± 1.5) and night 3 (8.7 ± 1.4), but no interactions were observed.

### Vigilance monitor

**Fig. 3:**
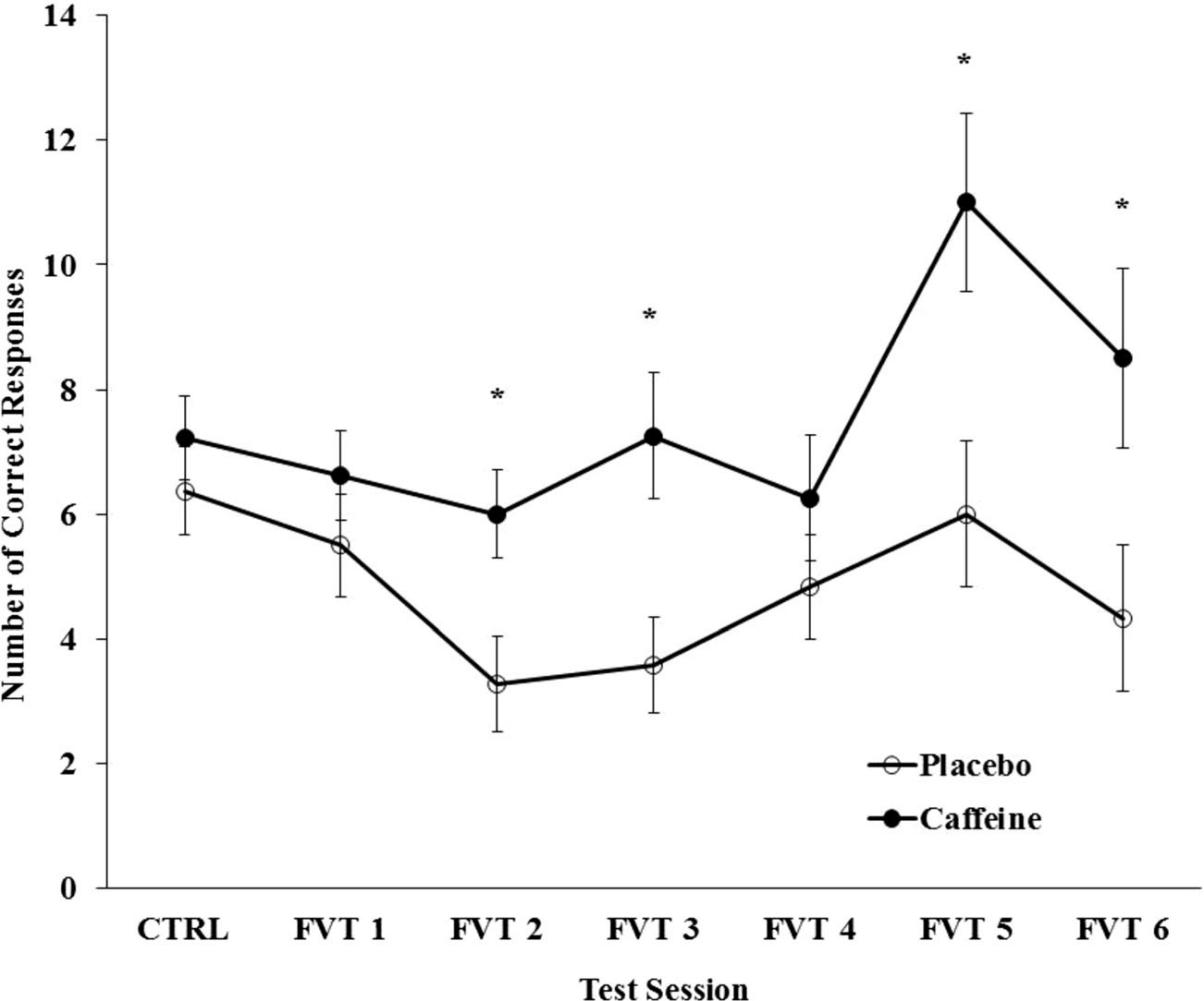
The number of correct responses to the stimuli presented on the vigilance monitor during the control (CTRL) and six field vigilance test (FVT) sessions for the caffeine and placebo groups. The *asterisk* indicates a significant difference between groups during the given test session

Overall, there was a main effect of the caffeine (*p* < 0.001) where the number of correct stimuli responses for participants receiving caffeine (7.6 ± 0.4) was significantly greater than those receiving placebo (4.8 ± 0.3). Figure 3 displays the number of correct responses over the test sessions. Post hoc analyses of the significant group × session interaction (*p* < 0.01) revealed that group responses were equal during control testing, but differences were observed between groups during sessions 2, 3, 5 and 6.

### Logical reasoning test (LRT)

**Fig. 4:**
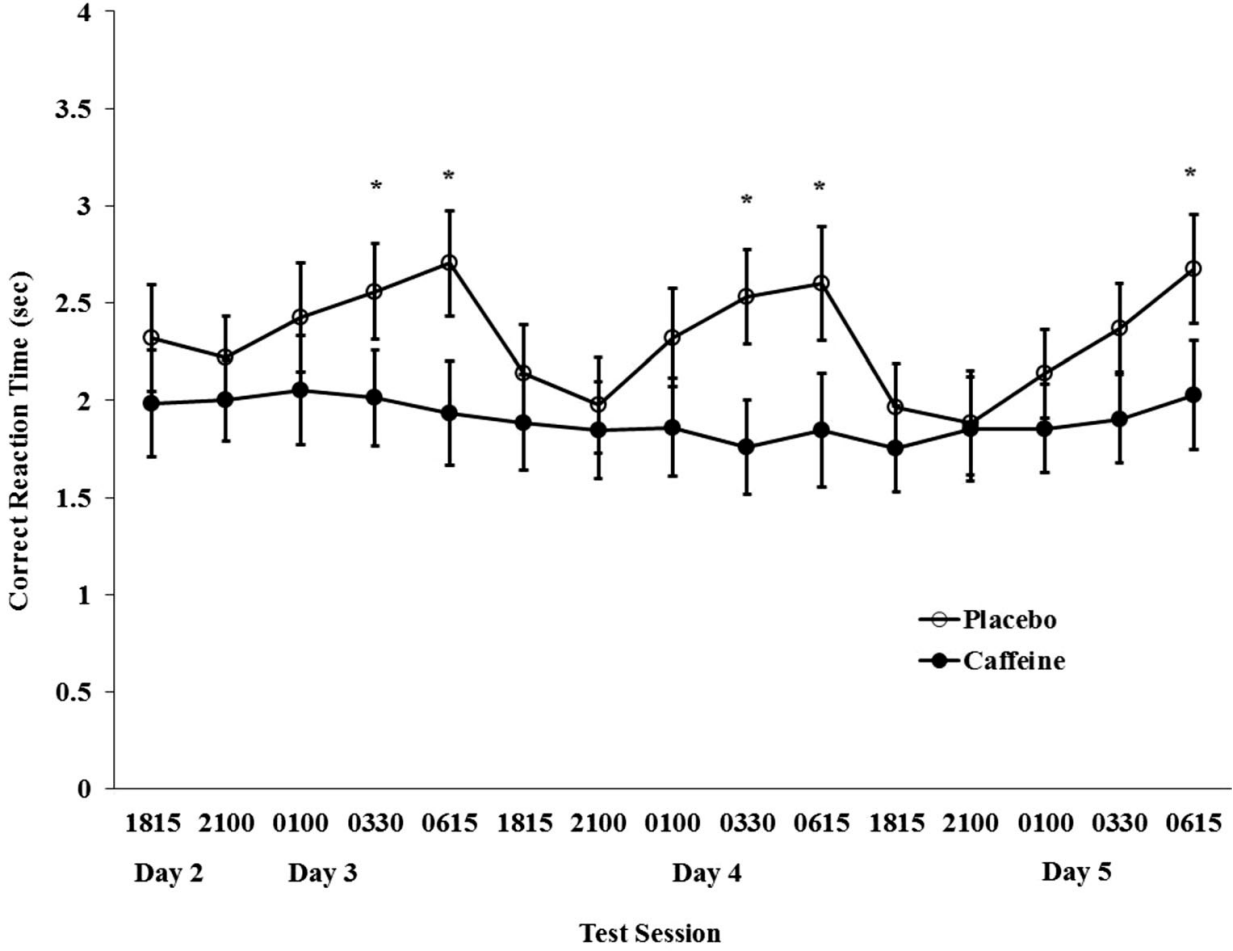
Mean correct response time during the logical reasoning test beginning during the evening of day 2 through to the morning of day 5 for the caffeine and placebo groups. The *asterisk* indicates a significant difference between groups during the given test session

Figure 4 presents the mean correct response reaction times over the test sessions. There was a main effect of group (*p* < 0.001) indicating that overall the caffeine group correctly responded more rapidly (1.9 ± 0.07 s) than placebo (2.5 ± 0.07 s). Post hoc analyses indicated there were no differences between groups at the beginning of each overnight period of testing. However, significant differences between groups were observed during the overnight trials at 0330 and 0615 h on days 3 and 4, and during 0615 h on Day 5 (*p* < 0.05).

There was a significant effect of treatment for the number of correct responses. Overall, participants in the placebo group made more correct responses (31.0 ± 0.2) than those receiving caffeine (30.0 ± 1.3). Post hoc analyses, however, did not reveal significant differences between groups at any of the test sessions.

### Live-fire marksmanship

Three participants were excluded from these analyses due to their lack of experience in marksmanship compared with the other volunteers. The caffeine and placebo groups consisted of nine and eight participants, respectively. There was no effect of treatment or interaction of treatment and test day on the percentage of foes correctly targeted. Similarly, there was no effect of treatment group or an interaction of treatment and test day on the percentage of friends correctly targeted.

### Relationship of history of caffeine use to overnight FVT

There was no relationship between history of caffeine use and performance during FVT compared with control scores during the overnight testing on day 3 (*r* = −0.01), day 4 (*r* = 0.02) or day 5 (*r* = 0.01) for those who received caffeine. Correlations were also non-significant for the placebo group, but values were moderately positive, indicating some relationship between a larger decrease in performance during FVT with greater daily caffeine use (*r* = 0.61, 0.46 and 0.32 for days 3, 4 and 5, respectively). However, these data were skewed by the one participant with the highest daily reported caffeine use of 410 mg. When their data were excluded, correlations again were non-significant and actually negative (*r* = −0.15, −0.12 and −0.60 for days 3, 4 and 5, respectively) suggesting that performance for those participants with higher daily caffeine use deteriorated less over the three nights. History of caffeine use was also not related to self-reported hours of sleep during FVT for participants in either group.

## Discussion

### Cognitive performance

This study demonstrates the effectiveness of caffeine as a countermeasure to offset impairments in cognitive function that accompany successive days of reduced sleep. In military theatres of operations, adverse changes in cognitive function are associated with increased friendly-fire incidents (Belenky et al. 1994), and in the transportation industry, these impairments are related to an increase in accidents (Horne and Reyner 1995). Importantly, this study suggests that the use of caffeine could sustain workplace productivity and safety in occupational settings that provide less than optimal periods of sleep during successive days of operations and require personnel to function during the overnight hours.

The impact of sleep loss on cognitive function is well documented (Dinges and Kribbs 1991; Kreuger 1989; Lieberman et al. 2002; Thomas et al. 2000), and the magnitude of these effects can be equal to or greater than the impairments associated with moderate levels of alcohol intoxication, clinical doses of sedating drugs or clinical hypoglycaemia (Lieberman et al. 2005). It was evident in this study that under such adverse circumstances reaction time slowed, vigilance decreased and speed of decision-making was reduced. This supports the general recommendation of optimising sleep behaviour in occupations where these cognitive components are important for ensuring safety in the workplace.

The benefits of using caffeine as a countermeasure to offset cognitive impairments during a period of sustained wakefulness are well established (Beaumont et al. 2001; Kamimori et al. 2000, 2005; Lieberman et al. 2002; Penetar et al. 1993; Walsh et al. 1990; Wesensten et al. 2005a). In a military setting, caffeine is also effective in maintaining vigilance and reaction time during periods of sleep deprivation (Lieberman et al. 2002; McLellan et al. 2005a, b), as well as restoring or improving physical performance (McLellan et al. 2004, 2005b, 2007) and maintaining or improving marksmanship (McLellan et al. 2005a; Tharion et al. 2003; Tikuisis et al. 2004). The findings from the current investigation extend these previous observations on the repeated use of caffeine during successive overnight periods of sustained operations with restricted opportunities for sleep.

Cognitive performance during the first overnight period of sustained wakefulness in the late evening of day 2 and early morning of day 3 was maintained with repeated 200-mg doses of caffeine as demonstrated by significant performance improvements in PVT, FVT and LRT compared with placebo. These differences between caffeine and placebo are consistent with our previous field studies that examined the effects of caffeine during a single overnight period of sleep loss (McLellan et al. 2005a, b). In addition, the current study demonstrated that subsequent 4-h sleep opportunities during the afternoon were not able to restore the impairments in cognitive function that followed the overnight periods of sustained wakefulness without caffeine supplementation. Those receiving placebo slept more during the overnight vigilance testing as well as during the afternoon sleep periods, yet their cognitive performance remained significantly impaired compared with those receiving caffeine. Despite obtaining less sleep during the afternoon periods, the soldiers’ cognitive performance improved with continued overnight caffeine supplementation and was comparable with baseline scores, with the exception of some reductions during the second overnight testing period.

It cannot be determined whether afternoon sleep periods assisted in maintaining cognitive performance for those receiving caffeine since our experimental design did not include a group that was required to remain awake for the duration of the study. Other studies that have included afternoon sleep periods prior to overnight sleep deprivation and compared the effectiveness of caffeine supplementation on cognitive function during the overnight period have used the prior sleep periods as a preventative measure (Bonnet and Arand 1994a; b; Bonnet et al. 1995; Schweitzer et al. 2006), rather than for the purpose of restoration following sleep loss as was performed in the current investigation. Many studies that have examined the effectiveness of caffeine alone to enhance cognitive performance during sleep loss have either tested the effect during a single overnight period of sustained wakefulness (Kamimori et al. 2005; McLellan et al. 2005a, b; Walsh et al. 1990) or did not provide the caffeine until a longer period (40 h or more) of sleep loss had elapsed (Kamimori et al. 2000; Lieberman et al. 2002; Penetar et al. 1993; Wesensten et al. 2002, 2004, 2005a). Thus, direct comparisons to the current investigation are not possible. However, during 48 h of sleep deprivation, it has been reported that repeated 300 mg doses of caffeine every 6 h beginning early during the first overnight period could not sustain cognitive performance at baseline levels during the second night of sleep loss (Bonnet et al. 1995). Similarly, although cognitive performance of the caffeine-treated group improved versus the placebo-treated group throughout 64 h of sustained wakefulness with twice daily doses of 300 mg slow release caffeine, performance began to deteriorate relative to baseline scores during the second overnight period of sleep loss (Beaumont et al. 2001). In the present study, there were also some decrements in cognitive performance for FVT during the second overnight period, but this was not the case for LRT, PVT or responses to the vigilance monitor and the FVT responses rebounded to baseline levels during the third night of testing. Thus, it appears that the additional sleep during the afternoon, albeit only 65 min as indicated by wrist monitors, was an effective strategy together with the repeated dosing of caffeine (total, 800 mg/night) during the overnight periods to sustain cognitive performance for the duration of the current study.

Interestingly, the combination of caffeine together with afternoon sleep periods maintained higher-order decision-making at baseline levels for the duration of the study. These effects are most clearly illustrated in Fig. 4 where the overnight circadian effect is demonstrated by the prolonged decision-making latencies during the LRT for the placebo group but not for those receiving caffeine. The positive impact of caffeine on decision-making processes has been demonstrated during the initial 24 h of sleep loss (Bonnet and Arand 1994b; Bonnet et al. 1995), but the effects become either less consistent (Wesensten et al. 2005a) or become similar to placebo with longer periods of sleep deprivation (Bonnet et al. 1995). Thus, in the present study, the additional afternoon sleep periods and/or the higher total dose of caffeine during the overnight periods may have accounted for the extended benefits that were observed during the LRT. Experimental design for future study would ideally include a group that was required to remain awake for the duration of the study in order to conclusively determine whether afternoon sleep periods assisted in maintaining cognitive performance for those receiving caffeine.

Occupational circumstances in the civilian workplace would generally be less extreme than the sleep-loss scenario examined in the current study, as the former might include additional nights of sustained wakefulness but longer recovery sleep periods during the day. Nevertheless, it has been shown that reducing daily sleep from 9 h to 5 or 7 h over a 7-day period produces persistent reductions in cognitive function during the day. The sustained impairment in cognitive function for the placebo group in the current study is consistent with these reductions in cognitive performance observed over this 7-day period of chronic sleep restriction (Belenky et al. 2003). Use of a countermeasure strategy, such as caffeine, would seem necessary to restore cognitive function, even in these less severe occupational settings, as long as the daytime recovery sleep periods are less than optimal. However, one might expect that the caffeine dose required to restore cognitive performance to baseline levels would be less than the cumulative dose of 800 mg used in the current investigation.

It should also be noted that continued overnight dosing of caffeine could compromise and/or reduce the duration and quality of the limited sleep opportunities that exist during the day. If quality of sleep is reduced, then performance benefits from caffeine could be reduced during subsequent overnight periods of sustained wakefulness. Studies that have assessed the impact of caffeine on quality of sleep have typically administered the drug during a period of sustained wakefulness (from 24 to 64 h), which was followed by a single recovery sleep period during the day (Beaumont et al. 2005; Carrier et al. 2007, 2009; Killgore et al. 2008; LaJambe et al. 2005; Wesensten et al. 2005b). Interestingly, those studies that assessed cognitive function following this recovery sleep period reported little, if any, impairment due to reduced quality of sleep following caffeine ingestion (Beaumont et al. 2005; Killgore et al. 2008; LaJambe et al. 2005; Wesensten et al. 2005b). However, none of these studies continued to assess cognitive performance during subsequent overnight periods of sleep loss that included caffeine ingestion, with additional recovery sleep periods provided during the day. In the present study, despite receiving less sleep during the afternoon recovery sleep periods (as indicated by actigraphy) the caffeine group continued to perform better during the overnight periods of sustained wakefulness. Thus, at least for the scenario of three successive overnights sessions, caffeine was a successful countermeasure strategy to sustain cognitive function despite the reduced duration of afternoon sleep. We cannot state for certain whether continued redosing with caffeine during additional overnight periods of sleep loss would continue to sustain cognitive performance despite potentially less recovery sleep. Also, with continued overnight dosing, a tolerance to caffeine may develop, which could reduce the effects of a given dose of the drug on cognitive function.

### Marksmanship

Accurate targeting of friend and foe is a skill requiring both fine motor control and higher-order decision-making capability. It has been reported that live-fire accuracy was impaired with sleep loss yet maintained with an overnight total caffeine dose of 600 mg when conventional forces personnel were tested (McLellan et al. 2005a). In addition, with highly trained Special Forces personnel, neither sleep loss nor ingestion of a cumulative 600-mg dose of caffeine affected live-fire marksmanship (McLellan et al. 2005b). In the current investigation, accurate live-fire decisions of friend and foe targeting were unchanged compared with placebo conditions over cumulative sleep loss. It is critical that caffeine not impair marksmanship if it is to be used in military settings where accurate marksmanship is essential. Fine motor control and hand steadiness may be reduced following ingestion of larger caffeine doses approximating 400 mg (Jacobson et al. 1991), and these changes might be expected to impact marksmanship accuracy. In this study, when testing followed the initial 200 mg dose of caffeine provided in the late evening on each day, accuracy was unchanged. It is not known whether testing conducted following the overnight periods of sleep loss that accompanied total caffeine doses of 800 mg, (approximately 10 mg kg^−1^) would have led to impairments in marksmanship. Although we did not directly assess hand steadiness, collectively, the findings suggest that caffeine will not have detrimental effects on live-fire marksmanship for highly trained military personnel. This observation is consistent with studies that assessed marksmanship with small arms simulators (Tharion et al. 2003; Tikuisis et al. 2004) rather than using live-fire, friend–foe testing scenarios.

### Limitations

There were certain study limitations that could affect the interpretation of our findings. First, experimental control is reduced during field experimentation, and access to study participants and time constraints negated options for inclusion of caffeine or placebo study groups who did not receive afternoon sleep periods. Nonetheless, the ecological validity of the study design, which included input of the end-user, is an important consideration with field experimentation, and reduced experimental control may be one limitation that cannot be fully addressed.

Second, outcome measures for cognitive tests such as the PVT and LRT involve speed or time. It has been suggested that caffeine can have a direct effect on nerve conduction velocity (Bazzucchi et al. 2011), which could account for some of the changes observed for the caffeine group with these dependent measures. In contrast, others have not observed an increase in motor unit firing rates following caffeine ingestion (Kalmar and Cafarelli 1999; Meyers and Cafarelli 2005; Plaskett and Cafarelli 2001), and these findings are consistent with our view that caffeine impacts cognitive function, as assessed by tests such as the PVT and LRT, through its antagonistic effects on central adenosine receptors rather than through a direct effect on nerve conduction velocity. Nevertheless, the reader should remain cognizant of the fact that caffeine’s effects on peripheral motor responses may influence outcome measures of tests designed to assess cognitive function.

Also, it has been suggested that caffeine’s positive effects on arousal and vigilance reflect primarily the reversal of withdrawal effects imposed by abstinence periods within study designs (James 1994; James et al. 2005; Rogers 2014). However, other investigators have disputed this hypothesis (Einöther and Giesbrecht 2013; Lieberman et al. 2002). In the present study, the abstinence period was almost 48 h prior to the first drug administration; thus, withdrawal effects, if present, should have begun to decrease (Juliano and Griffiths 2004). In addition, the majority of participants would be considered light or non-habitual consumers of caffeine where withdrawal effects would be minimal or non-existent (Rogers et al. 2013). Also, except for the one participant with the highest reported daily caffeine use, correlations between changes in performance during FVT over successive nights of testing and habitual caffeine use were weak and non-significant. Finally, the data were reanalyzed with the highest consumers of caffeine removed from both groups, and the main effect of the drug and the interaction effects remained as stated in the results. Interestingly, when only the low consumers (less than 50 mg day^−1^) were compared for the caffeine (*n* = 6) and placebo (*n* = 5) groups, the FVT still revealed an effect of the drug and a decrease in performance for the placebo group on nights 2 and 3, whereas the caffeine group’s performance remained unchanged. As a result, it is unlikely that withdrawal reversal was responsible for the differential effects observed between the drug and placebo groups.

The LRT demonstrated significant effect in which the placebo group made more correct responses, but overall the caffeine group correctly responded more rapidly. This suggests speed/efficiency of processing was improved with caffeine. In the Marksmanship test, response time was not measured; hence, it is not known whether placebo treated personal took longer to identify and accurately respond to the friend–foe stimuli resulting in the unchanged results in both groups for targeting.

Finally, cognitive function is known to exhibit a circadian rhythm (Monk et al. 1997), and the control FVT scores were obtained at a time where cognitive performance would be expected to be better than performance in the early morning hours. Thus, in addition to the effects of sleep loss, some of the deterioration in performance observed among the placebo group could reflect this circadian effect, which was also evident during the LRT (see Fig. 4). Nonetheless, caffeine was able to maintain performance during FVT comparable to the control condition throughout the overnight period of testing (see Fig. 2), and the drug removed any evidence of a circadian effect on the response to the LRT (see Fig. 4).

## Conclusion

This study demonstrates that 800 mg of caffeine provided during overnight periods of wakefulness is an effective strategy to sustain cognitive function when less than optimal sleep periods are provided during the afternoon. Reaction time, vigilance and logical reasoning were all maintained with caffeine supplementation and remained at or close to control levels for the duration of the study. Thus, when individuals are required to work during the overnight and do not receive sufficient sleep during the day, caffeine supplementation at night should be considered to help maintain workplace productivity and safety.
